# Mirror versus parallel bimanual reaching

**DOI:** 10.1186/1743-0003-10-71

**Published:** 2013-07-09

**Authors:** Farnaz Abdollahi, Robert V Kenyon, James L Patton

**Affiliations:** 1 Rehabilitation Institute of Chicago, Sensory Motor Performance Program (SMPP), Suite: 1406, 345 E. Superior Street, Chicago, IL 60611, USA; 2 University of Illinois at Chicago, Chicago, IL 60607, USA

**Keywords:** Bimanual training, Bimanual coordination, Self-telerehabilitation, Upper limb

## Abstract

**Background:**

In spite of their importance to everyday function, tasks that require both hands to work together such as lifting and carrying large objects have not been well studied and the full potential of how new technology might facilitate recovery remains unknown.

**Methods:**

To help identify the best modes for self-teleoperated bimanual training, we used an advanced haptic/graphic environment to compare several modes of practice. In a 2-by-2 study, we compared mirror vs. parallel reaching movements, and also compared veridical display to one that transforms the right hand’s cursor to the opposite side, reducing the area that the visual system has to monitor. Twenty healthy, right-handed subjects (5 in each group) practiced 200 movements. We hypothesized that parallel reaching movements would be the best performing, and attending to one visual area would reduce the task difficulty.

**Results:**

The two-way comparison revealed that mirror movement times took an average 1.24 s longer to complete than parallel. Surprisingly, subjects’ movement times moving to one target (attending to one visual area) also took an average of 1.66 s longer than subjects moving to two targets. For both hands, there was also a significant interaction effect, revealing the lowest errors for parallel movements moving to two targets (p < 0.001). This was the only group that began and maintained low errors throughout training.

**Conclusion:**

Combined with other evidence, these results suggest that the most intuitive reaching performance can be observed with parallel movements with a veridical display (moving to two separate targets). These results point to the expected levels of challenge for these bimanual training modes, which could be used to advise therapy choices in self-neurorehabilitation.

## Background

Bimanual training is particularly important in fostering recovery in neural injuries resulting in hemiparesis such as stroke, because the non-affected arm can potentially retrain the affected arm [1-3]. Upper extremity movements such as buttoning a shirt or zipping a jacket are simple but essential actions that need to be regained for making progress towards motor recovery and regaining activities of daily living (ADL). And although many studies focus on perfect performance, patients most care about task completion with proper coordination of both arms [4]. Therefore, bimanual therapy, which gives the users the possibility of achieving their primary goal of retraining ADL’s, should be of major importance to the therapist.

Several studies have demonstrated that bimanual training improves coordination between the paretic and non-paretic arms [5-11]; while a few have shown unwanted outcomes, such as reductions in the Fugl-Meyer or Ashworth scales (See [12,13] for a review). This discrepancy in performance may be due to the various ways the two limbs interact and move during bimanual training; for example, there are mirror-symmetric movements with respect to the body midline and asymmetric/alternating flexion-extension movements among others. Additionally, rigid coupling of the limbs (locking the actions of one limb to the other) often present in bimanual training could enable the paretic limb to act passively and depend mainly on the less affected limb for control, thus reducing its experience of the forces and motions associated with a particular movement. This may be why Kadivar *et al*., 2011 found no significant differences in bimanual performance (i.e. trajectory error) when one arm was rigidly forced to follow the other in parallel and mirror modes [14]. Thus, it remains unclear which methods are optimal.

Placing a subject in an environment that manipulates the visual feedback may help to resolve this discrepancy in the literature. Virtual environments can allow each arm to perform independently while presenting novel visual feedback. This promotes active participation of both limbs, and hence each limb is making and learning from its own mistakes. Furthermore, using this paradigm may tell us which form of uncoupled bimanual practice provides superior results i.e. mirror vs. parallel mode. Previous robotic rehabilitation studies that used the mirror mode of bimanual practice showed a significant increase in brain activation in similar parts of both brain hemispheres as well as enhanced inter-hemispheric activation [1,2,9,15-18]. However, to our knowledge, the *intuitiveness*, in terms of how fast and how accurate people can perform in this mode of practice, compared with the parallel mode, has not been studied in the uncoupled condition.

With two hands involved in practice, there are several approaches for coordinating both limbs. One is based on symmetry -- either transfer actions (parallel motions such as transferring a large object) or joint-similar actions (mirror motions such as opening a book). Lewis and Byblow reported that patients respond better to bimanual practices that involve in-phase and symmetric actions also denoted as mirror movements [19-21], which has been attributed to simultaneous brain activation of bilaterally homologous areas during these activities. However, these activations are not necessarily associated with functional gains, and the performance in a parallel mode was shown to be superior to mirror in a triangle drawing task [22]. Hence, it remains to be seen whether parallel or mirror modes might be superior in terms of trajectory error and/or task completion time with the limbs decoupled.

Besides muscle grouping and coordination, visual attention also plays a role in task difficulty in targeted reaching. Virtual reality displays allow the possibility of transforming one of the hand’s feedback to the opposite side, so that subjects only need to attend to one side of their view. We hypothesize that such a “one-target” visual transformation might reduce task difficulty over managing a divided view to two targets.

The present study used healthy individuals in a virtual environment to examine how these modes of bimanual practice influence performance on a simple reaching task. We investigated how different modes, symmetry and feedback, might influence performance and rate of learning (change of performance across time). Specifically, we focused on differences in bimanual reaching due to mirror vs. parallel arm movements. We investigated the performance of uncoupled, bimanual point-to-point reaching under four conditions; mirror reaching to one target (the “one-target” visual transformation), mirror reaching to two targets, parallel reaching to one target, and parallel reaching to two targets. This study showed lowest completion times and trajectory errors for parallel movements reaching to two targets, identifying the least challenging mode for bimanual practice, which may suggest the most appropriate mode for self-therapy in future neurorehabilitation interventions.

## Methods

### Participants

Twenty healthy right-handed individuals (12 male, age range 19–53, mean age 28 ± 9) with corrected 20/20 vision were invited to participate and consented using approved Institutional Review Boards from both Rehabilitation Institute of Chicago and University of Illinois at Chicago guidelines for protection of human subjects Internal Review Boards according to the declaration of Helsinki. A pilot study determined the effect size and inter-group variance to be 1.21 and 1.41 seconds, leading us to a power estimate of 5 subjects in each of four treatment groups (described below) based on Cohen’s method for an ANOVAs with a targeted power of 0.8 and significance levels of 0.05. Participants were naïve to the apparatus and had no history of previous musculoskeletal or neurological injury. The handedness of each individual was assessed using the modified Edinburgh Handedness Inventory [23]. Subjects were excluded if they scored less than 90 percent on the right-handedness test or if they had depth perception impairment of less than 8 out of 9 on graded circle test (Stereo Optical Company, Chicago, IL, USA).

**Figure 1:**
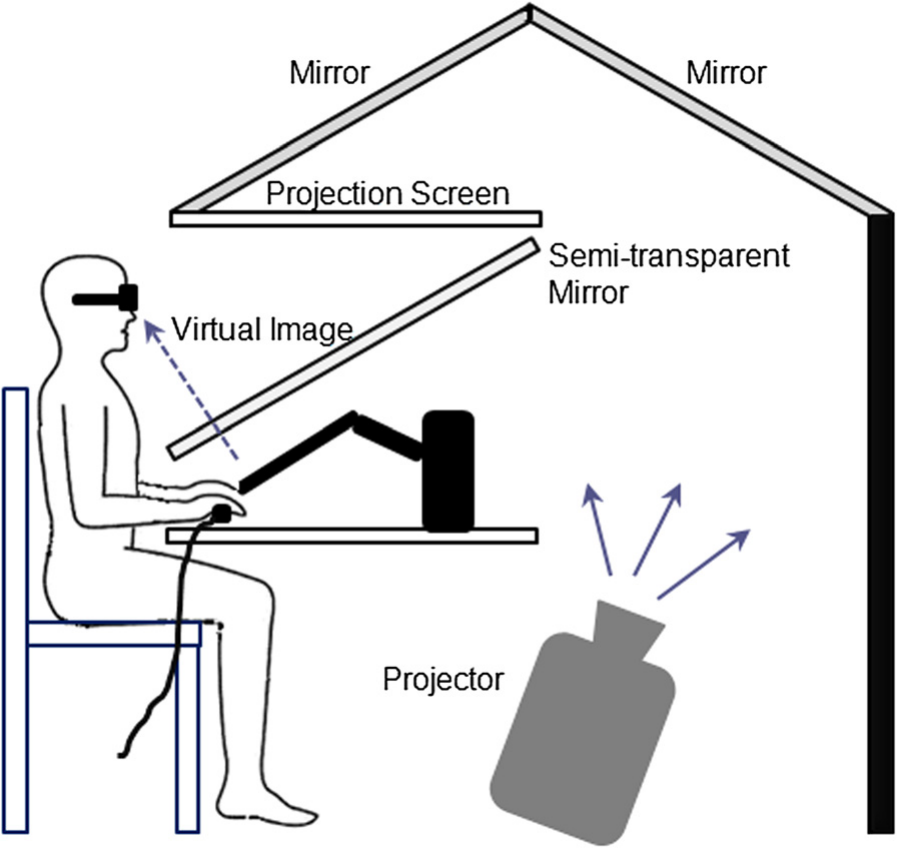
Experimental setup (VRROOM).

All experiments in this study were performed in a three-dimensional, large-workspace haptics/graphics system called the Virtual Reality and Robotic Optical Operations Machine (VRROOM; Figure 1) [24]. A cinema-quality digital projector (Christie Mirage 3000 DLP) displays the stereo images that span five-foot-wide 1280x1024 pixel display resulting in a 110º wide viewing angle in a see-through augmented reality display. In this study, vision of the arms was occluded so that only cursors (representing hand locations) and targets were shown. Infra-red emitters synchronize separate left and right eye images through LCD shutter glasses. Ascension Flock of Birds™ magnetic sensors tracked motion of the head to track the head position and re-render the environment when necessary so that the subject had the proper real-time view angle. Another sensor served as the position tracker of the right hand. A 6-degree of freedom PHANTOM Premium 3.0 robot (SensAble Technologies) provided tracking of the left hand.

### Experimental protocol

**Figure 2:**
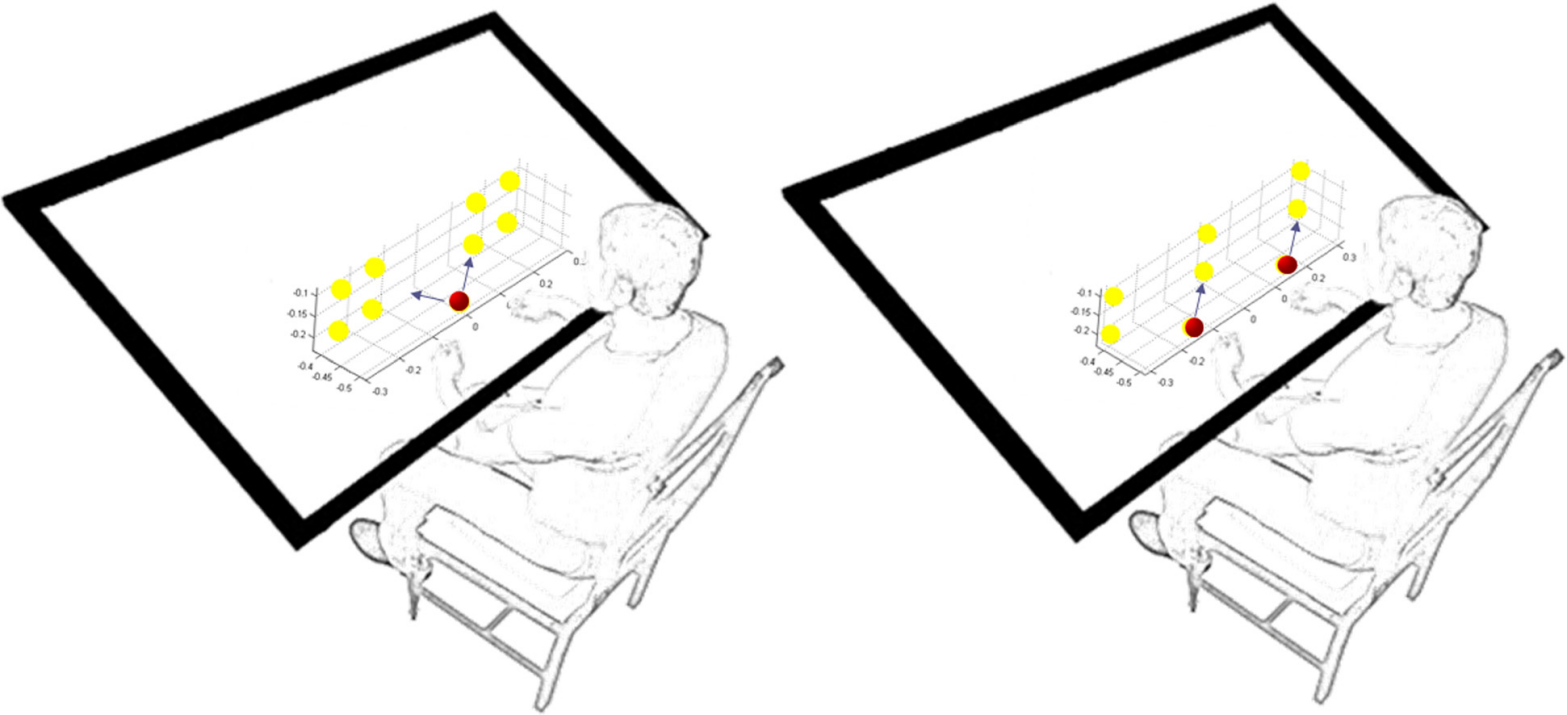
**Four different target locations per hand were presented in a random order with coming back to the home target** (**red**) **every time**; **Mirror** (**left**) **and Parallel** (**right**); **the arrows show the movement pattern in each group**; **the numbers represent the x**, **y and z coordination of the targets.**

Subjects were seated in a chair in front of the VRROOM. Hand position for left and right hands were obtained using a PHANTOM robot in left hand and a Flock of Birds position tracker in the right hand. These instruments are highly precise devices making it safe to assume they had similar accuracy and signal-to-noise ratios. Hand position data were sampled at 100 Hz. The PHANTOM robot exerted no forces during the experiment. Targets were displayed in the virtual environment such that the average distance that both hands were required to travel remained the same (Figure 2). Targets were placed to avoid crossing the midline in one of four randomly chosen locations, and were displayed on the screen as a sphere that the subject was instructed to move the cursor inside of. Alternating trials were at the initial position to ensure repeatable task requirements during training. All subjects were instructed to make straight and fast movements from the initial target to the final target. A movement (or trial) was considered complete when both cursors arrived at the appropriate target and halted for 0.5 seconds. Upon completion, the target(s) would vanish and the next target in the sequence would appear.

**Figure 3:**
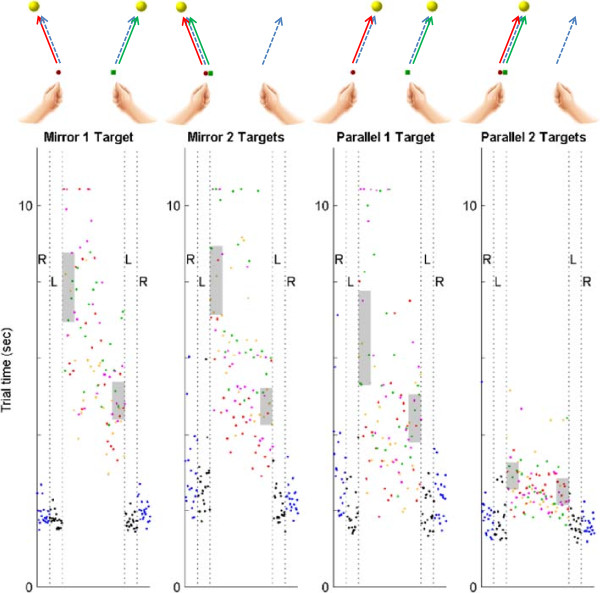
**Group description (top); hand movement (dashed arrow), cursor movement (solid arrow).** Sample learning curves for movement time (bottom); right hand (blue), left hand (black), bimanual (each color shows a different reaching direction), blocks used for data analysis (gray).

Participants were divided into four separate groups in a 2-by-2 design. Each group experienced one of the bimanual movement modes (either mirror or parallel) and one of the target requirements (move to either one or two targets) in a single session. For the one-target condition, the right hand’s cursor was transformed to be near the left, with the goal of having the cursors representing each hand moving side by side (Figure 3, top). This required subjects to visually attend to one area in the workspace. The remaining groups were required to move towards two targets while experiencing veridical feedback about the location of each hand, but had to attend to two areas (each) at the same time – the “two target” groups.

For the purposes of familiarization and to check the effects of bimanual movements on unilateral performance, subjects began with 40 unilateral movements per hand to randomly placed targets before and after the bimanual task. Each session consisted of 200 bimanual practice trials; 100 of these trials were center-out reaches to randomly placed targets. These outward trials were evaluated for their performance.

### Evaluation procedure

Because movement speed and accuracy are believed to intimately tradeoff [25], we assessed both movement time and trajectory error as primary measures of interest. Each trial’s movement time was calculated from the time that both cursors left the home position until the time they both entered their target radius and remained there for 0.5 sec. Each trial’s trajectory error was summarized using the typical measure of maximum perpendicular distance to the ideal line to the target [26].

Learning curves were plotted for all trials, but the above-mentioned measures were calculated only for the first and last 20 movements in the practice phase (gray shaded area, Figure 3, bottom). Repeated measures ANOVA was performed on both measures with main (between) factors being movement type (mirror vs. parallel) and number of targets (one vs. two) and the within factors being location of targets and different evaluation times in each trial. Statistical alpha levels were 0.05 to detect significance.

## Results

**Figure 4:**
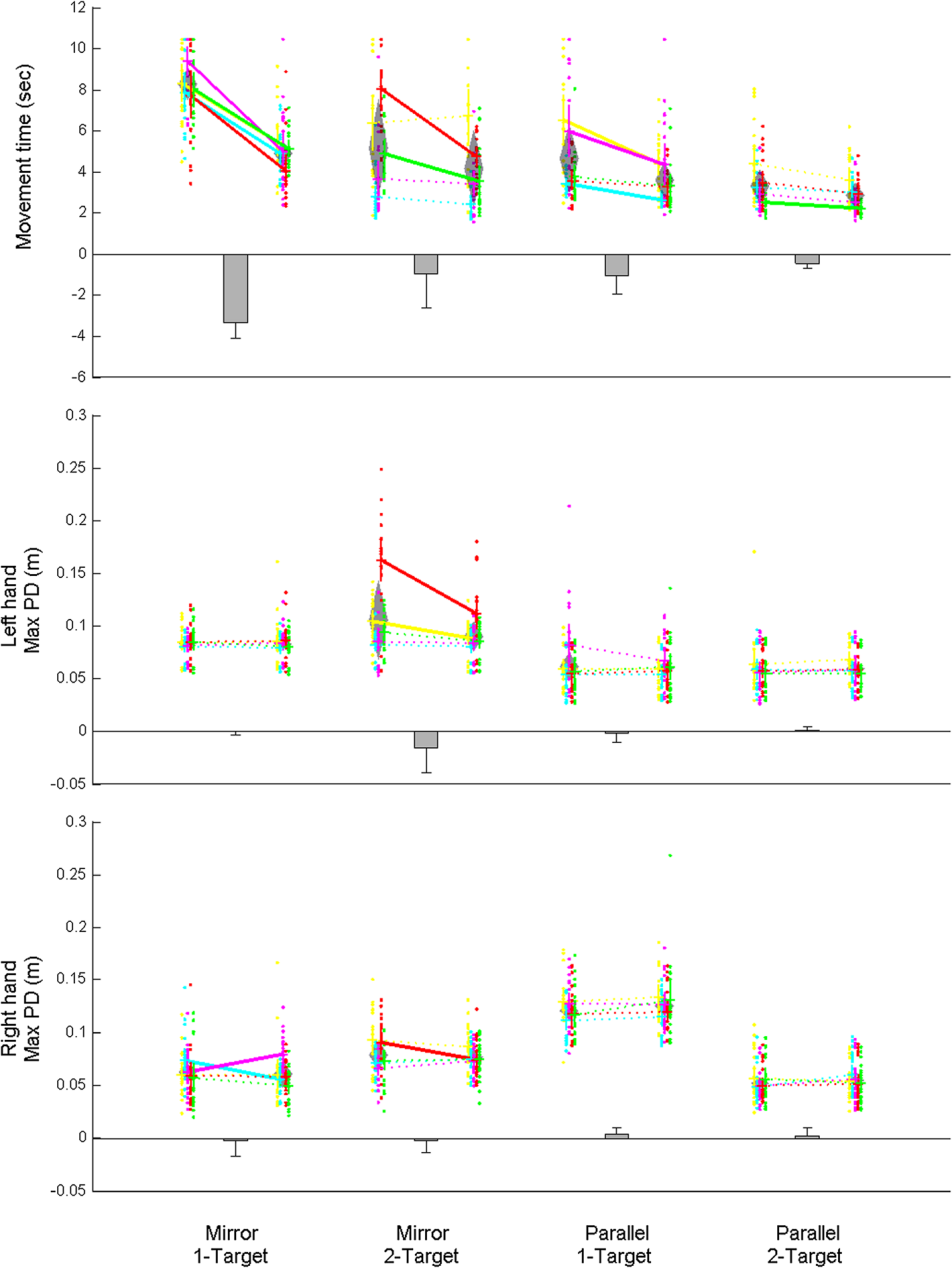
**Parallel two target group is the clear winner for both movement time and error.** Each column of dots represents a subject's 20 initial (lefthand) and 20 final (righthand) practice trials, with vertical lines indicating 95% confidence intervals. Change is indicated by diagonal lines (for subjects) and grey bars (group). Significance (solid lines), no significance (dash lines); subject (color).

The key findings of this study were that movement time and trajectory error were lowest for subjects reaching to two separate targets in parallel (Figure 4). Movement time was significantly lower for groups reaching in parallel (F(1,16) = 16.53, p < 0.001) and for groups reaching to two targets (F(1,16) = 8.94, p < 0.01). Trajectory errors were lowest for the parallel two-target group, indicated by a significant interaction effect between movement type and number of targets for both hands (F_right_(1,16) = 130.45, p < 0.001 and F_left_(1,16) = 39.37, p < 0.001).

Movement times changed least across practice for the parallel two-target group, indicated by a significant interaction amongst movement type, number of targets and practice (F(1,16) = 5.03, p < 0.05). Movement time changed an average of 1.43 seconds less across practice for the groups reaching in parallel, indicated by a significant interaction between movement type and practice (F(1,16) = 12.86, p < 0.01) (Figure 4, top). Furthermore, movement time changed an average of 1.49 seconds less across practice for groups reaching to two targets, as indicated by a significant interaction between number of targets and practice (F(1,16) = 14.07, p < 0.01). As Figure 4 (top) shows, the parallel two-target group begins with low movement time and exhibits a “floor effect” where there is little opportunity for improvement beyond their initial movement time [27].

Trajectory error results differed from movement time results. There was no significant change in trajectory error across practice for 16 of the 20 subjects from the beginning to the end of trials within each group (individual t-test, Figure 4, indicated by dashed lines). The right hand trajectory errors changed an average of 8 mm less across practice for the groups reaching in parallel, indicated by a significant interaction between movement type and practice (F(1,16) = 5.16, p < 0.05). The parallel two-target group showed a lower average error for both hands compared to all other groups even after training (Figure 4, middle and bottom). Finally, different target locations did not significantly affect movement time or trajectory errors.

**Figure 5:**
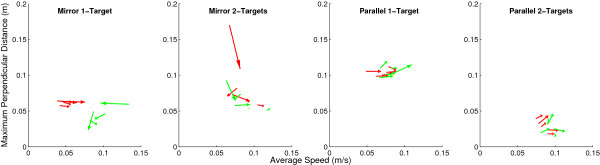
**Across practice, most subjects maintained left hand (red arrows) error while increasing speed in all groups.** Right hand (green arrows) error was maintained only in groups reaching in parallel mode. The tail and head of each arrow represents a subject’s average speed/accuracy combinations at the beginning (trials 1–20) and end (trials 80–100) phases of practice.

Further insight can be derived by inspecting how speed and accuracy interact across practice. Most subjects’ left hands increased speed while error remained constant (Figure 5, red arrows point to the right). Slopes of these red arrows were not significantly different from zero (p > 0.8). Right hands showed no particular trend.

## Discussion

This work shows that there are significant differences between how subjects perform bimanual targeted-reaching tasks under differing visual feedback modes. Among the four groups tested, the mode that involved parallel reaching to two targets clearly showed the lowest errors and shortest completion times at the beginning and throughout the experiment. In repetitive practice, errors did not tend to change across trials. Subjects tended to maintain lower left hand than right hand errors while increasing the average speed (and reducing completion times) of the left. While this paper does not attempt to understand any underlying neurophysiological processes, it reveals behavioral evidence that can inform choices in future bimanual applications.

The small change in both completion time and trajectory error observed in the group performing parallel reaching to two targets suggested that there was little (if any) learning. This lack of change maybe due to a floor effect because error was low at the start and throughout the trials. In addition, we speculate that this is the most familiar or intuitive mode of bimanual activity making it easiest for subjects to execute. This is consistent with “directional compatibility,” where limbs are more coordinated when endpoint directions agree [22]. We also speculate that other modes were more difficult, making them initially less familiar, fostering learning, and leading to improvement across training. This was especially dramatic for the mirror transformation, which had the largest errors, slowest completion times, but showed the largest amount of change (learning) across practice. Nevertheless, no other groups’ final errors were as low as the mode involving parallel reaching to two targets, suggesting that this mode is, by far, the most intuitive.

One issue not investigated in this initial study is the persistence of any learning effects. Depending on the bimanual training application, retention may be required at different times. Hence, the appropriate time for follow-up tests and the durability of learning should be evaluated in future use of our results in a particular application.

Our results differ from a related study by Kadivar *et al*., 2011, in which no difference between bimanual parallel and mirror modes was found. Our results, which detected significant differences, may have been due to differences in task between these two studies -- their task coupled the limbs through a robotic interface, while ours allowed each hand to move independently. Our study also calculated error differently -- we used maximum perpendicular distance from the line to the target, while Kadivar and colleagues averaged this distance and divided by path length. Such dividing by path length can mask error. For example, a movement with several reversals might result in a deceptively low value if divided by its long length. Our data showed similar trends for both of these measures, but normalized average error produced more variable results.

Contrary to our assumption that attending to only one visual target area would simplify the task, we observed longer movement times in the “one target” modes that involved cursor transformations. This poor performance may result from the subject’s need to reinterpret or mentally transform the conflict between the movements of the hands and its associated visual feedback [28-30]. Such conflict may place a further attentional burden that lengthens completion time. Furthermore, of these two transformed modes (one target), mirror feedback showed longest completion times of all and hence was deemed the most difficult. Although participants in this group significantly improved in movement time, the final performance was still not as good as other groups even after 200 trials. The remaining “two target” feedback modes performed significantly better, which suggests that attending to two different visual areas is easier than mentally transforming visual cues. This separation of targets to different areas of visual space may also involve parallel computations in separate somatotopic areas of visual cortex that require less competing neural resources [31,32]. Also, such visual transformations are not commonly encountered in the physical world, while simultaneous attention to two areas is a frequent ecological challenge to humans in tasks such as typing, drawing, and playing video games [26]. Parallel modes, now possible with such virtual reality technology, may provide the most intuitive feedback for training environments.

Our mirror (one target) approach also differed from approaches that use physical mirrors to display limb actions [33-36]. In previous mirror approaches, reflection of one hand replaced the visual feedback of the other. Here, we transformed the right hand cursor so that it appeared on the same side as the left cursor, which we speculate to be more challenging. Such a mirror transformation could provide a “feedback puzzle” that may promote learning. Such complex challenges may encourage recovery better than intuitive ones [37], but these more challenging tasks might also be discouraging to some individuals. Hence, the results of this study serve merely as a guide to identify training modes that are either challenging or intuitive.

Nearly all participants kept error constant across training while decreasing completion times (with the exception of one subject with very high initial error). Participants improved speed rather than accuracy, which is one choice in the scheme of speed-accuracy tradeoff (Fitts’ law) [25]. Some have shown increasing speed in the course of learning a skill [38], while others have shown error reduction [39]. Therefore, changes in speed or accuracy may depend on the task. Interestingly, each group’s error was maintained at a different level. We speculate that each bimanual task requires its own level of information processing until a competent strategy is learned. Therefore, subjects hold error constant, begin slowly and speed up as they train. It remains to be seen whether these error levels reflect physiological limits in sensorimotor pathways or simply a different “tolerance” for error in each feedback condition.

These results have implications in rehabilitation, where bimanual interactions can assist a person in re-learning movement skills [40]. Our results suggest that parallel reaching to two targets may be the optimal method for such self-telerehabilitation because it is the most familiar (least challenging) mode of practice. To the patient, however, improvement in bilateral symmetry may not be as important as completing a functional tasks, some of which are asymmetric. It is also possible that intuitive modes for healthy may not be equally intuitive for brain injured individuals. It remains to be seen whether these results translate effectively to neurorehabilitation. Nevertheless, the initial findings presented here in healthy subjects can help identify environments for rehabilitation or in any training situation requiring bimanual practice.

## Abbreviations

ADL: Activities of daily living; VRROOM: Virtual reality and robotic optical operations machine; PARIS: Personal augmented reality immersive system.

## Competing interests

The authors have declared that they have no competing interests.

## Authors’ contribution

FA, RVK and JLP have made substantial contributions to conception and design. FA and JLP have been involved in interpretation of data. FA has been involved in acquisition of data, analysis and drafting the manuscript. JLP and RVK have been involved in revising the manuscript critically for important intellectual content. All authors have given final approval of the version to be published.
